# CRISPR-Cas9-mediated mutagenesis of the *SlSRM1-like* gene leads to abnormal leaf development in tomatoes

**DOI:** 10.1186/s12870-021-03397-5

**Published:** 2022-01-03

**Authors:** Yao Tang, Huijia Li, Chunxin Liu, Yuqing He, Hexuan Wang, Tingting Zhao, Xiangyang Xu, Jingfu Li, Huanhuan Yang, Jingbin Jiang

**Affiliations:** College of Horticulture and Landscape Architecture, Northeast Agricultural University, Harbin, 150030 Heilongjiang Province China

**Keywords:** SlSRM1-like, Leaf development, Auxin, CRISPR/Cas9, Tomato

## Abstract

**Background:**

Leaves, which are the most important organs of plants, can not only fix carbon sources through photosynthesis, but also absorb nutrients through transpiration. Leaf development directly determines the growth, flowering and fruiting of plants. There are many factors that affect leaf development, such as the growth environment, gene expression, and hormone synthesis. In this study, tomatoes were used to study the role of the transcription factor *Solanum lycopersicum* salt-related MYB1-like (SlSRM1-like) in the development of tomato leaves.

**Results:**

Loss-of-function of the *SlSRM1-like* gene mediated by clustered, regularly interspaced, short palindromic repeat (CRISPR)/CRISPR-associated 9 (Cas9) resulted in abnormal tomato leaf morphology, including thinner leaves, wrinkled edges, raised veins, disordered edge veins, and left and right asymmetry. An analysis of the transcription levels of genes related to leaf development revealed that the expression of these genes was significantly altered in the *SlSRM1-like* mutants (*SlSRM1-like*-Ms). Moreover, the *SlSRM1-like* gene was expressed at higher transcription levels in young tissues than in old tissues, and its expression was also induced in response to auxin. In addition, the transcription levels of genes related to the auxin pathway, which regulates tomato growth and development, were severely affected in the *SlSRM1-like*-Ms. Therefore, it is hypothesized that the *SlSRM1-like* gene functions in the regulation of tomato leaf development through the auxin-related pathway.

**Conclusions:**

In this study, we successfully knocked out the *SlSRM1-like* gene in the tomato variety Ailsa Craig using CRISPR technology and found that knockout of the *SlSRM1-like* gene resulted in abnormal development of tomato leaves. Further research indicated that *SlSRM1-like* regulated tomato leaf development through auxin-related pathways. The results provide an important reference for the functional study of other *SRM1-like* genes in plants and provide new insights into the regulation of leaf development in tomato and other plants.

**Supplementary Information:**

The online version contains supplementary material available at 10.1186/s12870-021-03397-5.

## Background

As signaling molecules, small amounts of plant hormones can exert substantial effects [1]. Plant hormones participate in the regulation of all aspects of plants—from seed germination to fruit ripening and even plant withering—through their synthesis, transport and degradation [2–4]. Auxin was the first plant hormone to be discovered, and studies have shown that auxin regulates the growth and development of plant vegetative and reproductive organs at many different stages and auxin is the most important and direct factor that affects the development of plant organs [5–8]. In addition, auxin can also interact with other hormones in response to specific stimuli [9, 10].

Leaves are important plant organs and are mainly responsible for nutrient synthesis through photosynthesis and osmotic pressure regulation, which depends on transpiration [11, 12]. Therefore, leaf development directly affects the growth, fruiting and reproduction of plants [13]. There are many factors that affect leaf development, generally through regulation of the plant’s own gene expression and growth environment [14, 15]. As an endogenous hormone that regulates growth and development, auxin strongly affects the development of different tissues of leaves at different periods [16]. In *Arabidopsis thaliana* plants, not only cotyledons but also young leaves and even expanding leaves have the ability to synthesize auxin, thereby controlling the expansion of leaves according to precise changes in auxin concentrations [17]. The methylation of indoleacetic acid (IAA) alters the homeostasis of auxin, causing the leaves of *Arabidopsis* to curl [18]. At the proximal axis of leaf primordia, auxin is actively effluxed through polar transport to reduce auxin concentrations, which affects the polar-related morphology of leaves [19]. MONOPTEROS (MP) expressed in the adaxial direction can directly bind to the promoters of the *WUSCHEL-RELATED HOMEOBOX* (*WOX*) genes *WOX1* and *PRS* to activate their expression in the leaf marginal region, thereby making the leaf flat [20]. The synthesis of auxin and its flow between cells have important effects on the development of leaf veins [21]. Specifically, *YUCCA* (*YUC*) genes play important roles in the development of leaf margins, which is also achieved through the effect of auxin [22]. In tissue culture-generated seedlings of *Nicotiana tabacum*, *Orychophragmus violaceus* and *Brassica chinensis*, the addition of auxin polar transport inhibitors resulted in the asymmetric growth of the leaves, indicating that auxin played a vital role in the process of symmetrical leaf growth [23]. Mutants of rice (*Oryza sativa*) have narrowed leaves, which is directly related to a decrease in auxin content in the leaves [24]. Another rice mutant, the *narrow leaf21* (*nal21*) mutant, exhibits obvious changes in leaf width, leaf length, leaf veins, and the size and number of epidermal cells, which is also caused by an abnormal auxin response because of the lack of function of the ribosomal small subunit protein RPS3A encoded by *NAL21* [25].

In tomatoes (*Solanum lycopersicum*), several genes that can affect leaf development have been reported, most of which are also related to auxin. Downregulation of *Lanceolate* (*LA*) gene expression causes the leaflets to become larger and the leaf margins to grow continuously, while *LA* overexpression causes the leaves to be lanceolate [26]. The *Lyrate* (*LYR*) gene can affect the sprouting of leaflet primordia and lateral growth of leaf margins, and its expression is positively regulated by auxin [27]. Overexpression of the *Petroselinum* (*PTS*) gene can inhibit interactions involving the *knotted*-like homeobox 1 (KNOX1) protein, thereby participating in the formation of compound leaves and increasing leaf complexity [28]. Loss-of-function of the *Regulator of axillary meristems1-like* (*RAX1-like*) gene leads to a decrease in the number of leaflets and leaf margin serrations, and the leaf margin becomes smooth [29]. The *Trifoliate* (*Tf*) gene can affect the formation of lobules of compound leaves and regulate the germination of meristems within leaf axils [30].

In *Arabidopsis*, the *Salt-Related MYB1* (*SRM1*) gene has been shown to be an important transcriptional regulator. First, it was observed that *SRM1* can affect the germination of seeds, and then, it was proven that *SRM1* can affect vegetative growth and leaf shape. Loss-of-function of the *SRM1* gene changes the morphology of rosette leaves in *Arabidopsis* and makes the leaves smaller, while the overexpression of the *SRM1* gene promotes the vegetative growth of the leaves [31]. However, the function of the *SRM1* gene in other species has not been reported. Here, we found that the loss-of-function of the *SlSRM1-like* gene, which is orthologous gene of the *AtSRM1* gene in tomato, severely affected the growth and development of tomato leaves. We used CRISPR technology to knock out the *SlSRM1-like* gene in tomato, observed phenotypic changes and detected the transcription level of related genes in the mutants. In addition, the *SlSRM1-like* gene was subjected to phylogenetic tree analysis and expression analysis in tomato. The results have reference significance for functional research on other *SRM1* genes, and provide new insights into the developmental mechanism of plant leaves.

## Results

### SlSRM1-like is a MYB-related transcription factor located in the nucleus

**Fig. 1:**
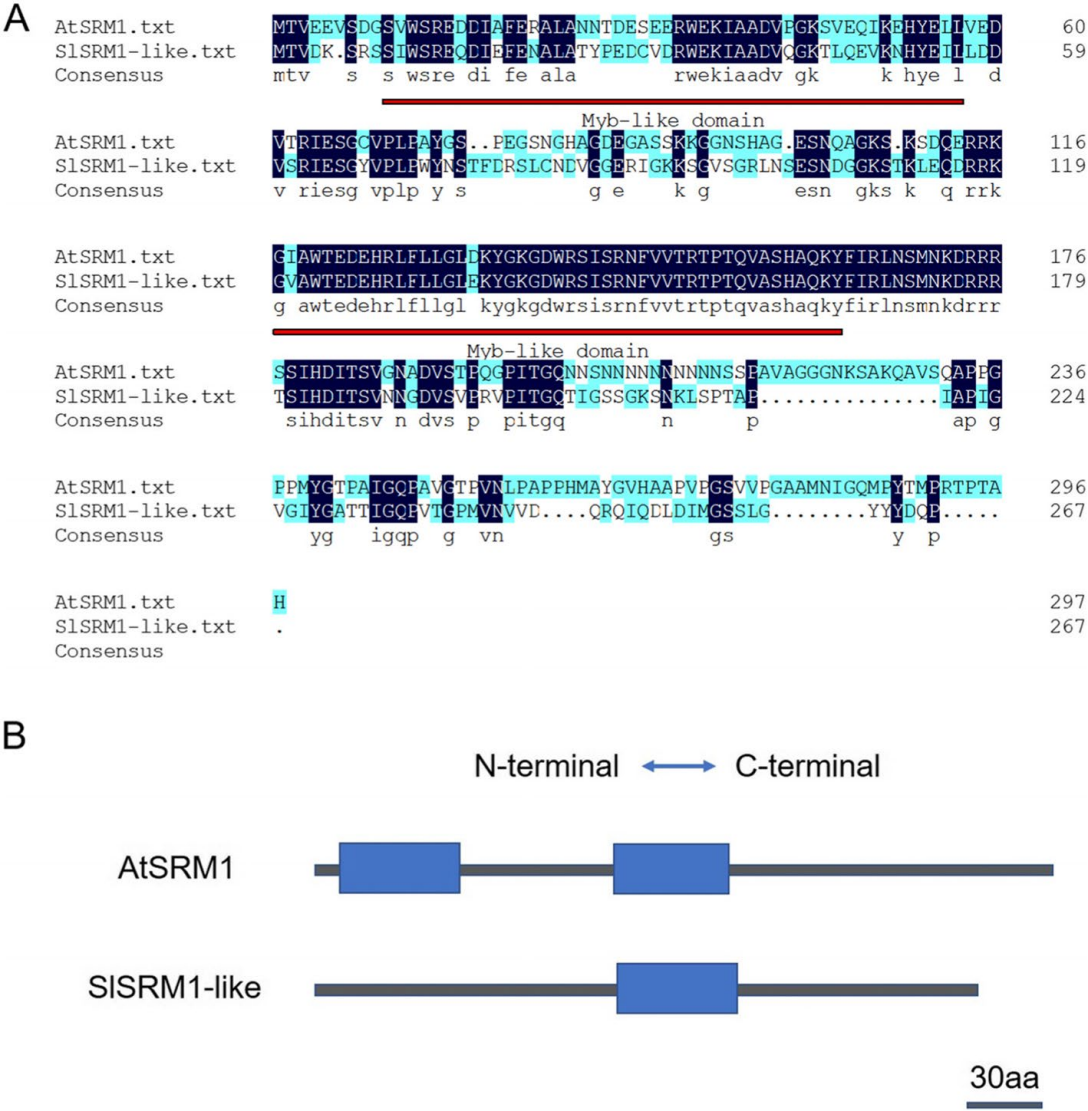
Comparison of SlSRM1-like and AtSRM1 proteins. **A** Amino acid sequence alignment of SlSRM1-like and AtSRM1 proteins. DNAMAN 6.0 software was used for sequence alignment. The parts with identical amino acid sequences are shown in dark blue and displayed on a separate line. The areas marked in red are Myb-like DNA-binding domains. The numbers on the right represent the number of amino acids. The numerical value of amino acid sequence similarity is not shown in the figure. **B** Position of the Myb-like DNA-binding domain in the SlSRM1-like and AtSRM1 proteins. The graph was constructed based on the prediction results of Pfam online software. The blue box represents the Myb-like DNA-binding domain, and the black line represents other amino acids connected to the domain. Left to right is the direction of the protein from the N-terminus to the C-terminus. The length and position of the blue box and black line correspond to the arrangement of amino acids in the protein. The length of the bar in the figure represents 30 amino acids

When we analyzed the transcriptome data of leaves from different cultivated tomato plants, we noticed the *Solyc04g008870.2.1* gene by chance. We compared its sequence with others via the Basic Local Alignment Search Tool (BLAST) and found that this gene was predicted to be SRM1-like, which is a member of the MYB-related transcription factor family. After DNAMAN alignment, the amino acid sequence identity of SlSRM1-like and AtSRM1 (At5g08520) was 50.99% (Fig. 1A). We then used Pfam online software to analyze the domains of the SlSRM1-like and AtSRM1 proteins. The results showed that the AtSRM1 protein contained two Myb-like deoxyribonucleic acid (DNA)-binding domains, while the SlSRM1-like protein contained only one Myb-like DNA-binding domain (Fig. 1B). The amino acid sequence alignment results showed that the regional sequence identity of the first Myb-like DNA-binding domain was not very high. Realignment of this region of the amino acid sequences revealed that the similarity was only 58.33%. This might be why this region was not identified as a Myb-like DNA-binding domain when Pfam online software was used to analyze the SlSRM1-like protein. Therefore, unlike AtSRM1, SlSRM1-like is a MYB-related transcription factor with one Myb-like DNA-binding domain.

**Fig. 2:**
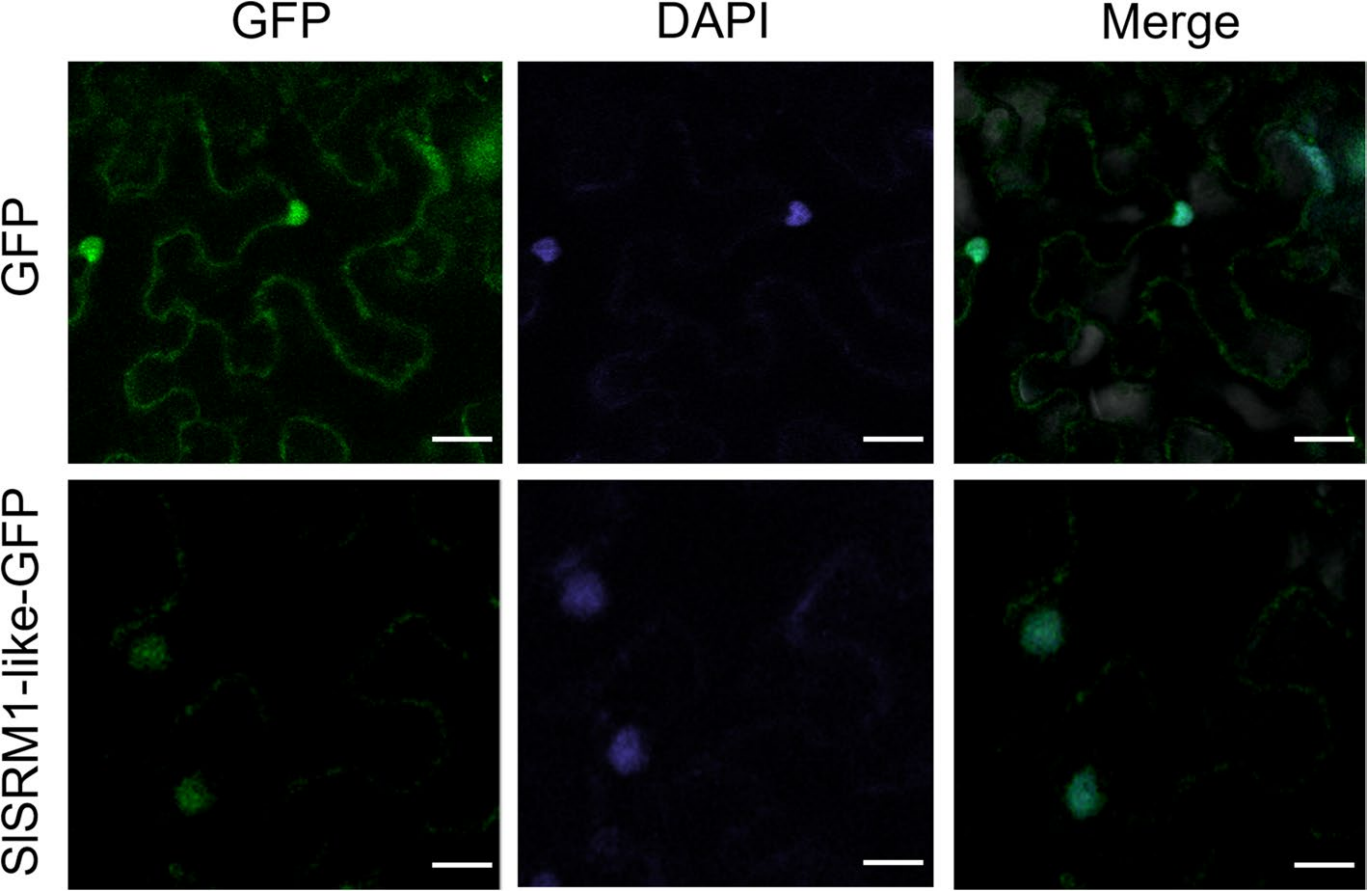
Localization of the SlSRM1-like-GFP fusion protein driven by the CaMV35S promoter in tobacco epidermal cells. The pYBA1132 vector was used in this experiment, and an empty vector was used as a control. *Nicotiana benthamiana* plants grown for 1 month were selected as materials. GFP, green fluorescent protein. Bars = 25 μm

As a transcription factor, SlSRM1-like functions in the nucleus and regulates the expression of corresponding target genes. To explore the location of the SlSRM1-like protein, we constructed a SlSRM1-like-green fluorescent protein (GFP) fusion protein for subcellular location observations. The results showed that the SlSRM1-like-GFP fluorescence appeared in the nucleus, indicating that the SlSRM1-like protein localized to the nucleus (Fig. 2).

### Orthologs of SlSRM1-like are widely distributed in plants

**Fig. 3:**
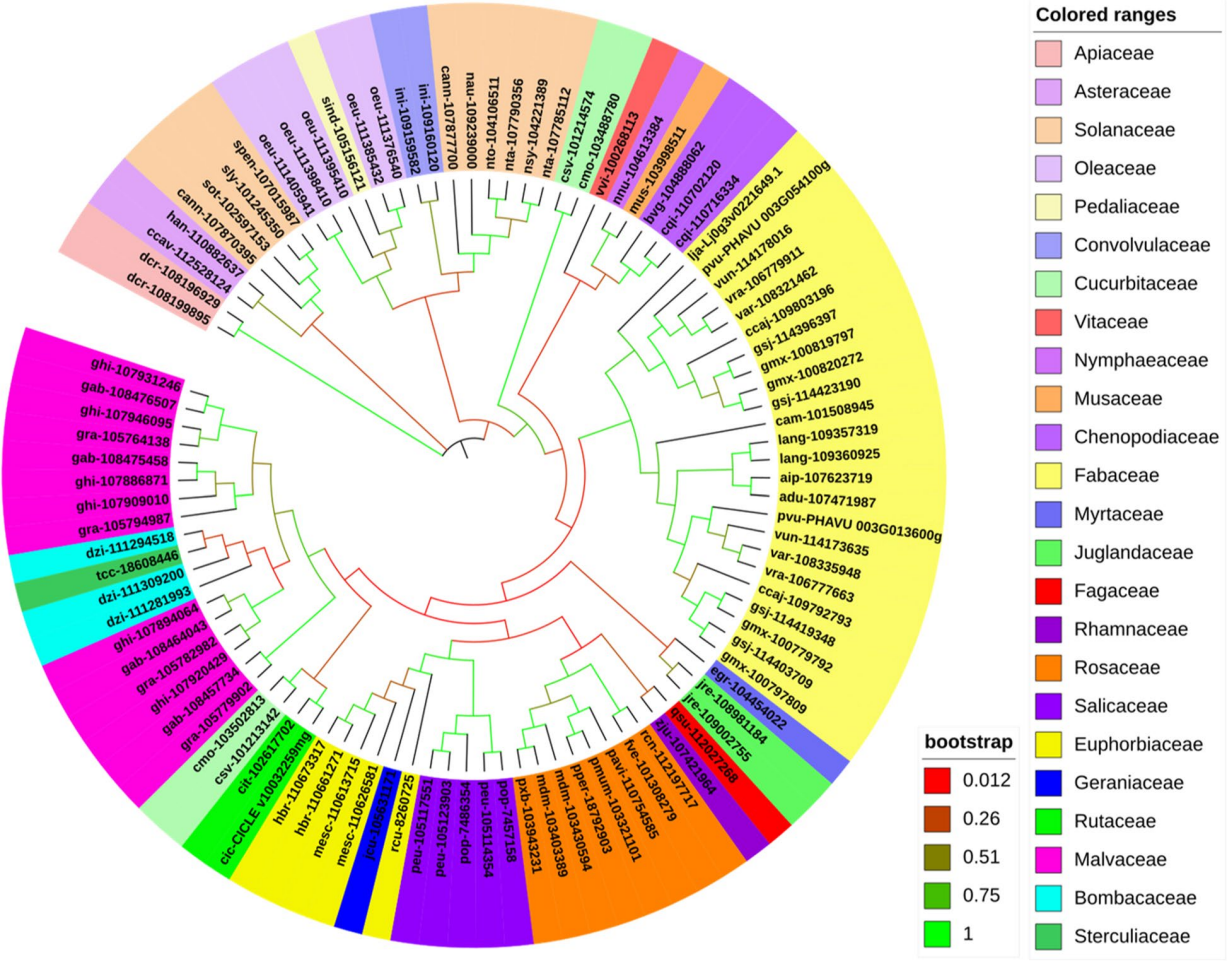
Phylogenetic analysis of the *SlSRM1-like* gene. The genes of plants belonging to the same family are marked with the same color. The names of genes involve a combination of two components. The letter before the short line is the species code, and after the short line is the gene name. The name of the *SlSRM1-like* gene in the figure is sly-101,245,350

We used the SlSRM1-like protein as the target protein for BLAST queries and found a large number of orthologs. The top 100 genes with high similarity were selected to construct a phylogenetic tree. The results showed that these 100 genes were associated with 57 kinds of plants in 24 different families, of which most were from Fabaceae. The SlSRM1-like protein (sly-101,245,350) was closely related to proteins in *Solanum pennellii*, *Solanum tuberosum*, and *Capsicum annuum*, which belong to the Solanaceae family (Fig. 3). Therefore, *SlSRM1-like* orthologs are widely present in a variety of plant species, and they are not unique genes in tomato species.

### The SlSRM1-like gene is expressed in multiple tissues, and its expression is induced by auxin

**Fig. 4:**
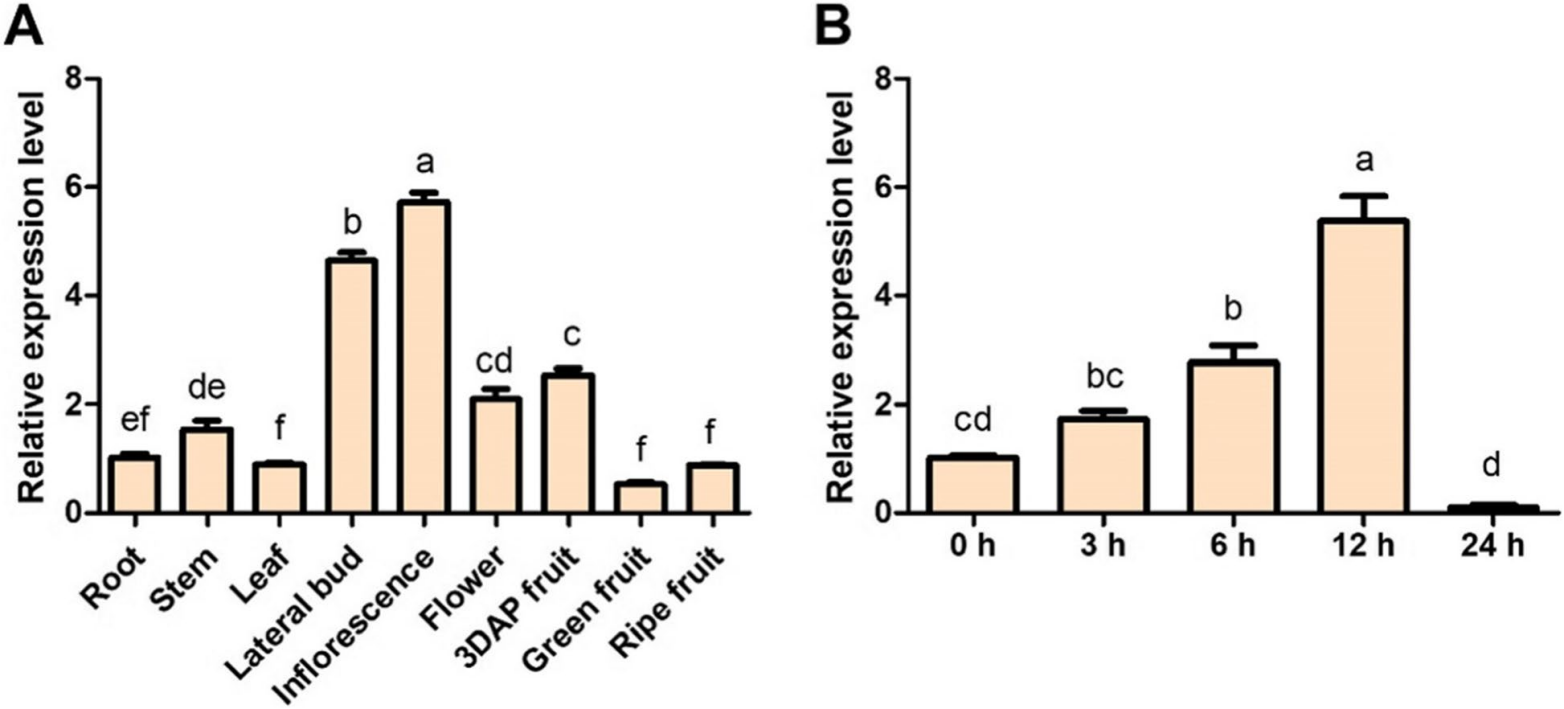
Characteristics of *SlSRM1-like* gene expression. **A** Expression of the *SlSRM1-like* gene in different tissues of tomato plants. Except for the fruits, all the tissues were collected from tomato plants at 45 d after sowing. DAP, days after pollination. Green fruit refers to the fruit before color change. The expression level in each tissue was based on the expression in the roots. **B** Expression changes of the *SlSRM1-like* gene over time after treatment with auxin. Thirty-day-old tomato plants were sprayed with 50 μmol/L IAA solution, and plants sprayed with distilled water were used as controls

To explore *SlSRM1-like* gene functions in tissues, we evaluated multiple tissues of the tomato plants. The results showed that the *SlSRM1-like* gene was expressed in both vegetative and reproductive tissues. The expression level of the *SlSRM1-like* gene was higher in young tissues, such as lateral buds and inflorescences, but lower in the roots, stems, leaves and other tissues of mature plants. In fruits, the expression of the *SlSRM1-like* gene was higher during the early stage of fruit formation (Fig. 4A). Therefore, we hypothesize that the *SlSRM1-like* gene is expressed in multiple tissues of tomato plants, especially in young tissues where the expression level is higher.

AtSRM1 could affect the shape of *Arabidopsis* leaves and promote vegetative growth. *SlSRM1-like* was also expressed in tomato leaves. Therefore, to explore whether the *SlSRM1-like* gene was related to the growth and development of tomato leaves, we subjected tomato seedlings to auxin treatment to detect whether the expression of the *SlSRM1-like* gene changed. The results showed that when the tomato plants were sprayed with auxin, the transcription level of the *SlSRM1-like* gene first increased and then decreased, peaking at more than 5 times the initial level after 12 h (Fig. 4B). Taken together, these results indicate that the expression of the *SlSRM1-like* gene is induced by auxin.

### CRISPR-mediated knockout of the SlSRM1-like gene leads to phenotypic changes in tomato leaves

The *SlSRM1-like* gene was highly expressed in young tissues, and its expression was also induced by auxin. Various characteristics prompted us to ask whether the *SlSRM1-like* gene was related to the growth and development of tomato organs. Therefore, we knocked out the *SlSRM1-like* gene and observe whether the appearance of the tomato plants would change.

**Fig. 5:**
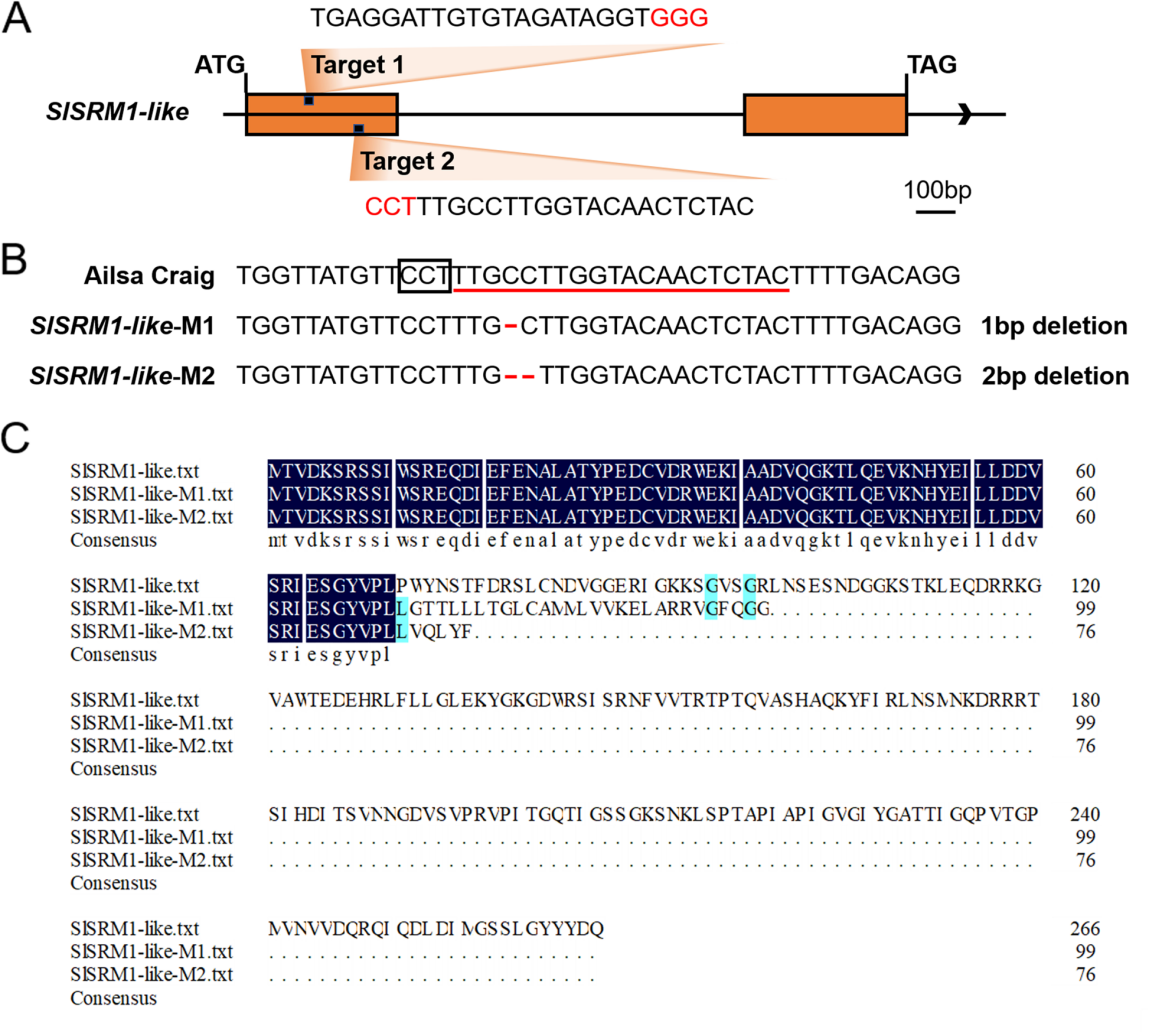
CRISPR/Cas9-mediated mutation of the *SlSRM1-like* gene. **A** Schematic diagram of the sgRNA target sites on the *SlSRM1-like* gene. The exons are indicated by yellow boxes, and introns and intergenic sequences are indicated by straight lines. The arrow indicates the direction of the genome, from 5′ to 3′. The black dots in the yellow box represent the sgRNA target sites whose sequence is shown next to them. The PAM motifs are marked in red. Bar = 100 bp. **B** Sequencing results of the sequence near the mutation site of the *SlSRM1-like* gene. The lower part of the sgRNA target site is marked with a red line, and the PAM motif is enclosed in a black box. **C** Amino acid sequence alignment of SlSRM1-like and SlSRM1-like-Ms proteins. DNAMAN 6.0 software was used for sequence alignment

We selected two target sites on the first exon of the *SlSRM1-like* gene as small guide ribonucleic acids (sgRNAs) to construct a vector for CRISPR (Fig. 5A, Fig. S1). Tissue culture-generated tomato plantlets infected by *Agrobacterium* and of the T_0_ generation with kanamycin (Kan) resistance were obtained. After the identification of *Kan* gene-specific primers, the polymerase chain reaction (PCR) results of 4 plants were positive (Fig. S2). Among these 4 tomato plants, 1-bp and 2-bp deletion mutations were found at the editing targets of the two plants, respectively (Fig. 5B). For the two potential off-target sites (SL2.50ch09: + 69,989,496; SL2.50ch02: − 48,992,078) in the coding DNA sequence (CDS) region predicted by the CRISPR-P 2.0 website, we did not detect any mutations in the corresponding regions (Fig. S1). In the T_1_ generation plants that were planted and cultured after harvesting the seeds from the T_0_ generation, we successfully screened mutant plants that were homozygous for the mutation site and did not have transfer DNA (T-DNA) insertion. The T_2_ generation plants obtained from the self-replication of T_1_ generation plants were used for further experiments.

**Fig. 6:**
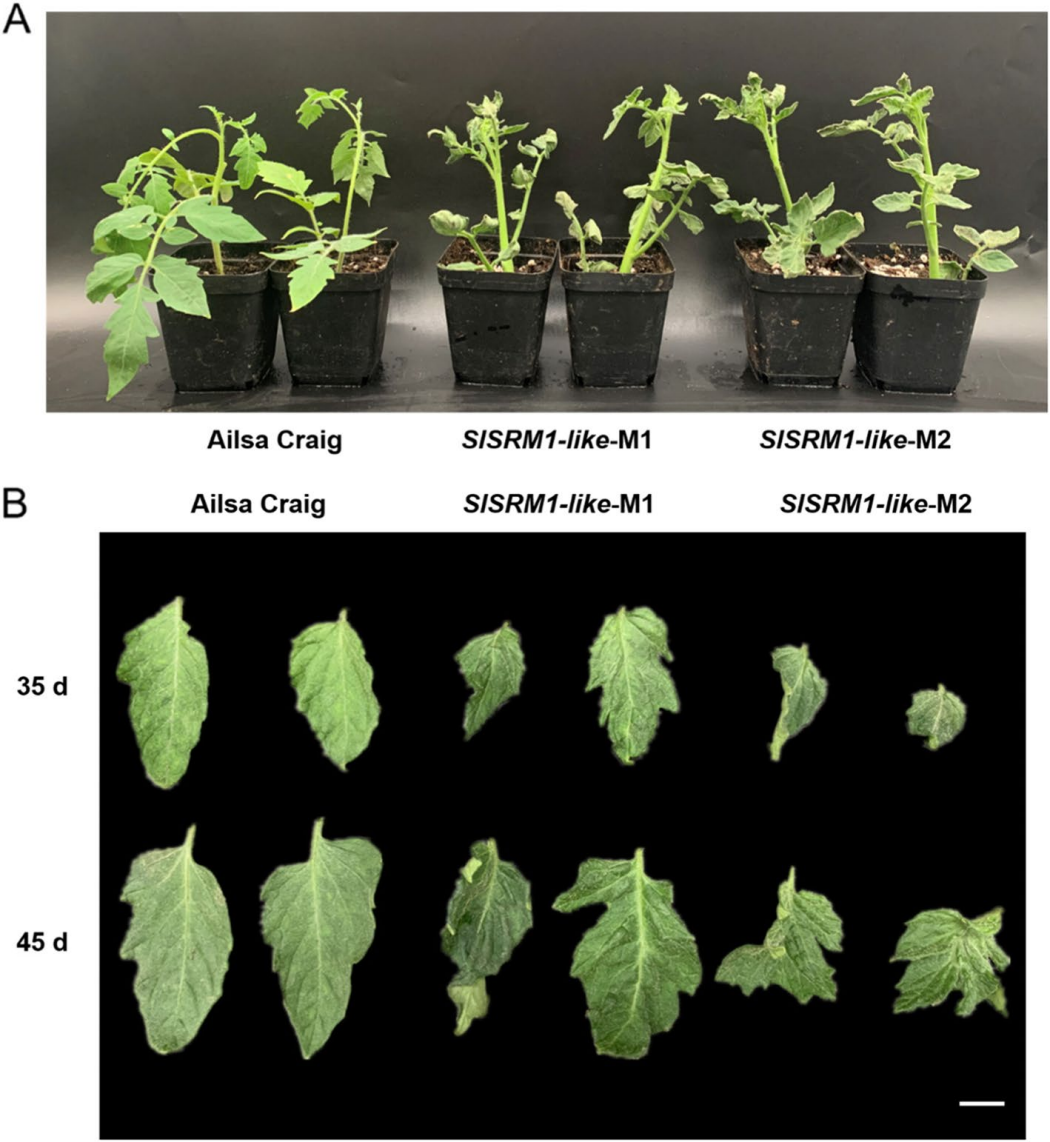
Morphological changes of leaves in *SlSRM1-like*-Ms plants. **A** Images of tomato seedlings. *SlSRM1-like*-Ms were T_2_-generation plants (Same below). **B** Image of leaves of tomato plants. The leaves in the first row were cut 35 d after sowing, and the leaves in the second row were cut 45 d after sowing. Bar = 1 cm

**Fig. 7:**
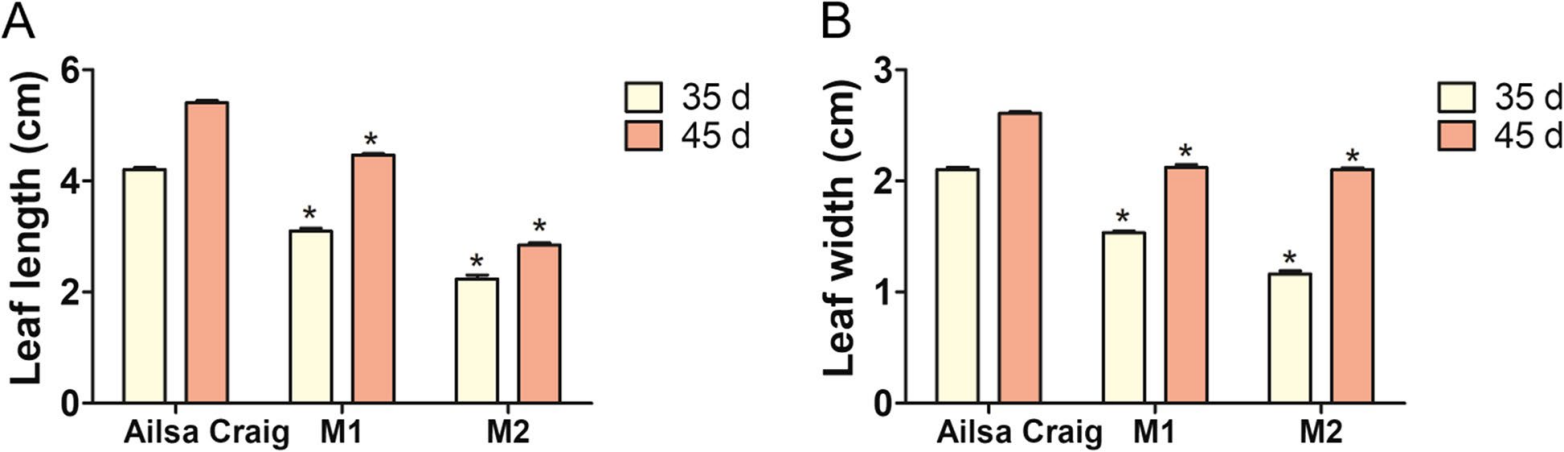
The statistical histogram of leaf size of *SlSRM1-like*-Ms compared with that of Ailsa Craig. Student’s *t* test was used for statistical analysis of leaf size (*, *P* < 0.05). **A** Leaf length. **B** Leaf width

Compared with those of Ailsa Craig plants, the leaves of CRISPR-mediated *SlSRM1-like* gene mutant plants (*SlSRM1-like*-M1 and 2) were substantially different. The leaf mesophyll tissue of the *SlSRM1-like*-M lines was not fully developed, and the leaves were generally very thin, causing the veins to bulge upward from the leaves. The convex leaf veins were distributed extremely unevenly, and the left-right symmetry of the leaf was disrupted. The branching of the leaf veins became irregular, especially at the ends, and the edges of the leaves presented strange shapes due to developmental disorders. Bending and wrinkling were also present in many leaves (Fig. 6A and B). Further sectioning results also showed that the leaf development of *SlSRM1-like*-Ms was incomplete and that the cell arrangement was significantly reduced compared with Ailsa Craig (Fig. S5). Moreover, the statistical results of leaf size showed that the leaf length and width of the *SlSRM1-like*-Ms were significantly reduced compared with those of Ailsa Craig (Fig. 7).

The 1-bp and 2-bp deletions in *SlSRM1-like*-M plants inevitably led to frameshift mutations of the corresponding protein. Amino acid sequence comparisons revealed that the SlSRM1-like-M1 protein changed beginning at the 71st amino acid, and the TAA stop codon appeared early (at the 100th amino acid position), which caused the protein to be truncated. The SlSRM1-like-M2 protein became shorter, consisting of only 76 amino acids (Fig. 5C). Moreover, the original Myb-like DNA-binding domain also disappeared from the truncated protein (Figs. 1 and 5C).

These findings confirm that the *SlSRM1-like* gene can affect the development of veins and mesophyll in tomato leaves.

### Transcript levels of genes related to leaf development changes in *SlSRM1-like* mutants

**Fig. 8:**
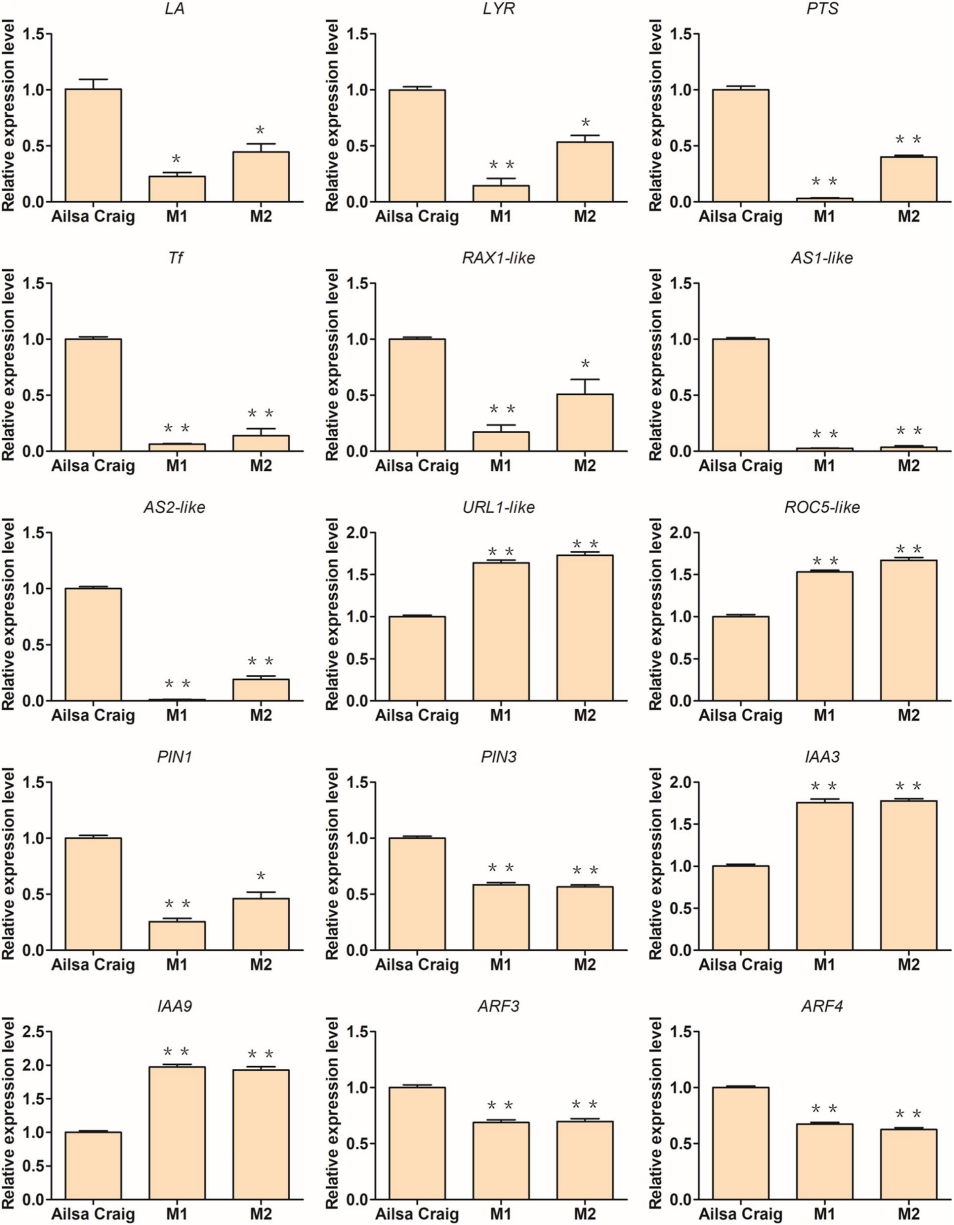
Analysis of the transcription of related genes in the *SlSRM1-like*-M mutants. Ailsa Craig plants served as controls. The leaves of tomato plants that were approximately 30 d of age were used for quantitative fluorescence experiments. *LA*, *Lanceolate*; *LYR*, *Lyrate*; *PTS*, *Petroselinum*; *Tf*, *Trifoliate*; *RAX1-like*, *Regulator of axillary meristems1-like*; *AS*, *Asymmetric leaves*; *URL1*, *Upward rolled leaf 1*; *ROC5*, *Rice outermost cell-specific 5*; *ARF*, *Auxin response factor*; *IAA*, *Indole-3-acetic acid*. Student’s *t* test was used to analyze the significance of the qRT-PCR results (*, *P* < 0.01; **, *P* < 0.001)

Since the leaves of *SlSRM1-like*-Ms plants had undergone substantial changes, we compiled a list of several genes reported to be involved in leaf development to detect whether their transcript levels changed. Compared with those in Ailsa Craig leaves, the transcript levels of *LA*, *Tf*, and *RAX1-like* genes, which are related to leaf margin development, in *SlSRM1-like*-Ms leaves significantly decreased. Loss-of-function of the *LYR* gene caused leaf tissue thinning, the *PTS* gene affected leaf shape, and the relative expression of these two genes also decreased in the *SlSRM1-like*-Ms leaves. In addition, the orthologs of *Asymmetric leaves 1* (*AS1*) and *Asymmetric leaves 2* (*AS2*), *Asymmetric leaves 1-like* (*AS1-like*) and *Asymmetric leaves 2-like* (*AS2-like*), which regulate the formation of symmetric laminae in *Arabidopsis*, were expressed at reduced levels in the *SlSRM1-like*-Ms leaves. *Upward rolled leaf 1* (*URL1*) and *Rice outermost cell-specific 5* (*ROC5*) control the rolling of rice leaves, and their orthologs *URL1-like* and *ROC5-like* were expressed at increased levels in the *SlSRM1-like*-Ms leaves. Moreover, we also analyzed some genes related to the auxin pathway, including *PIN-formed 1* (*PIN1*), *PIN3*, *Auxin response factor* (*ARF*) *3*, *ARF4*, *Indole-3-acetic acid* (*IAA*) *3*, and *IAA9*, and the transcription levels of these genes were significantly altered in the *SlSRM1-like*-Ms leaves (Fig. 8).

## Discussion

### SlSRM1-like plays an important role in regulating the development of leaf morphology and structure in tomatoes

The rise of CRISPR technology has provided a highly convenient way to study the functions of genes in animals and plants, especially for observations of tissue morphology and structure [32]. Gene editing technology that relies on the CRISPR/Cas9 system has been applied recently in tomatoes [33, 34]. To better study the function of the *SlSRM1-like* gene, it is necessary to obtain lack-of-function mutants through gene editing technology. Since the transcription level of the *SlSRM1-like* gene in young tissues was higher than that in older tissues and since its expression was also induced by auxin (Fig. 4), we wondered whether the function of the *SlSRM1-like* gene was related to the growth and development of tomato plants; thus, Ailsa Craig was chosen as the material for CRISPR operations.

Loss-of-function of the *SlSRM1-like* gene led to many different changes in the morphology of tomato leaves, such as prominent leaf veins, thinning of leaves, bending of leaf edges and left-right asymmetry (Fig. 6). Therefore, we searched for genes related to leaf development and investigated whether their transcription level changed in the *SlSRM1-like*-Ms plants. Among these genes, downregulated expression of the *LA* gene caused the leaflets of the compound tomato leaves to increase in size, and the leaf margins grew continuously [26]. The *LYR* gene affects the outward growth of the leaflet primordia and controls the lateral growth of the leaf margins [27]. The *RAX1-like* gene also regulates the development of tomato leaf margins, and its downregulated expression decreases edge serrations, resulting in smooth leaf edges [29]. The *Tf* gene inhibits the differentiation of leaf margins [30]. *URL1* and *ROC5* have been reported to be involved in the regulation of leaf rolling in rice [35, 36]. Therefore, we searched for their orthologs and investigated their transcription levels, and we found that these genes were expressed at higher levels in the leaves of *SlSRM1-like*-Ms (Fig. 8). Perhaps it was the altered expression of these genes that made the leaf margins of *SlSRM1-like*-Ms plants become curved and folded, with irregularly curved edge veins, which led to leaf edge deformities and even left-right asymmetry. In *Arabidopsis*, the *AS1* and *AS2* genes are related to leaf midrib development, and their downregulated expression causes the leaf veins to protrude from the leaves [37, 38]. We found orthologs of these two genes in tomatoes. The expression of these two orthologs in *SlSRM1-like*-M was also downregulated (Fig. 8), which might explain the prominent phenomenon of leaf midribs in *SlSRM1-like*-Ms plants. The *LYR* gene could also play a role in the development of laminar tissue [27], and its downregulated expression in *SlSRM1-like*-Ms might explain the thinning of the leaves of the mutants.

The *SlSRM1-like* gene can affect the development of many aspects of leaves, and it can also affect the expression of many genes that do not affect each other (Figs. 6 and 8). Perhaps the regulation of tomato leaf morphology by the *SlSRM1-like* gene occurs in early leaf development. The expression of the *SlSRM1-like* gene in young tissues was higher than that in old tissues, verifying this speculation (Fig. 4A). Furthermore, the *AtSRM1* gene can affect both the morphology of *Arabidopsis thaliana* leaves and seed germination [31]; that is, it already functions during seed germination. Perhaps the *SlSRM1-like* gene begins to function in the early stage, ultimately resulting in normal tomato leaf development.

### The function of the *SlSRM1-like* gene may depend on the auxin-related pathway

Auxin is a hormone closely related to the growth and development of plant tissues and generally has a higher content in young, actively growing tissues in the growth phase [39]. The expression of the *SlSRM1-like* gene in young tissues was higher than that in old tissues (Fig. 2B). It seems that there is a consistent trend between the expression of the *SlSRM1-like* gene and the content of auxin. Moreover, when tomato plants were sprayed with auxin solutions, the transcription level of the *SlSRM1-like* gene was obviously affected, first increasing and then decreasing (Fig. 4B), which proved that there is a close relationship between *SlSRM1-like* gene expression and auxin.

Aux/IAA proteins can inhibit the activity of ARF and are central to the regulation of auxin-mediated processes [40]. SlIAA3 and SlIAA9 have been shown to play an important role in tomato growth and development [41, 42]. In the process of auxin signal transduction, SlARF3 and SlARF4 also play an important role, affecting tomato development [43, 44]. PINs are responsible for auxin transport and have an important impact on the growth and development of tomatoes [45]. Therefore, we tested the expression levels of some genes related to the auxin pathway and found that the transcription levels of these genes changed significantly in the leaves of *SlSRM1-like*-Ms compared with Ailsa Craig (Fig. 8). Perhaps the deletion of the *SlSRM1-like* gene specifically affects the expression of these genes and eventually leads to tomato leaf abnormalities. In terms of the underlying reason, further experimental verification is needed.

### SRM1-like is a transcription factor widely found in plants

Transcription factors are a class of proteins that can bind to the promoter of a target gene to regulate the expression of that target gene at a specific time and intensity. Therefore, transcription factors are generally localized to the nucleus [46]. In this experiment, the SlSRM1-like protein was localized to the nucleus (Fig. 2), which is consistent with the characteristics of transcription factors.

Phylogenetic trees can reflect the genetic and evolutionary relationships between a protein and its orthologous proteins in other species [47]. Among the orthologous proteins of SlSRM1-like, the top 100 proteins with the highest homology were selected to construct a phylogenetic tree. These 100 proteins were associated with 57 species in 24 different families, and all the species are plants (Fig. 3), indicating that the *SRM1-like* gene is widespread in plants and that this gene is conserved.

## Conclusion

In this study, we found that the transcription factor SlSRM1-like plays an important role in regulating leaf development in tomatoes. First, knockout of the *SlSRM1-like* gene resulted in abnormal development of tomato leaves, such as thinning, wrinkling, bending, left-right asymmetry, and bulging veins. Moreover, the expression levels of genes related to leaf development changed significantly in the *SlSRM1-like*-Ms compared with those in the Ailsa Craig. In addition, the expression of the *SlSRM1-like* gene was induced by auxin, and the transcription level of genes related to the auxin pathway that regulates tomato growth and development was severely affected in the *SlSRM1-like*-Ms. Therefore, it is hypothesized that the *SlSRM1-like* gene functions in the process of regulating tomato leaf development through the auxin-related pathway. In summary, this study provides an important reference for the functional study of other *SRM1-like* genes in plants and provides new insights into the regulation of leaf development in tomato and other plants.

## Materials and methods

### Plant materials and growth conditions

Seeds of the wild tomato variety Ailsa Craig were provided by our laboratory. All the plants were cultivated in a plant growth chamber (16 h of light/8 h of darkness, room temperature) in a greenhouse at Northeast Agricultural University in Xiangfang district, Harbin, Heilongjiang Province. Normal water and fertilizer management practices were adopted for plant cultivation.

Tomato seedlings (30 d) were sprayed with IAA solution (50 μmol/L). Plants sprayed with distilled water were used as controls. The blades of the plants were removed 3, 6, 12, and 24 h after spraying and stored at − 80 °C. Tissue from 45-day-old plants was selected for quantitative real-time PCR (qRT-PCR) analysis. All the samples were collected from different plants exhibiting the same growth status, and at least 6 samples from the same plant were collected and reserved for future use.

### Analysis of gene sequences

Sequence data were retrieved from the National Center for Biotechnology Information (NCBI) website (https://www.ncbi.nlm.nih.gov/). The BLAST website (https://blast.ncbi.nlm.nih.gov/Blast.cgi) and DNAMAN 6.0 software were used for sequence alignment, and the Pfam website (http://pfam.xfam.org/) was used for the identification of protein domains.

### Total RNA extraction, cDNA synthesis and qRT-PCR

All RNA samples were extracted using a plant RNA extraction kit (TaKaRa MiniBEST Plant RNA Extraction Kit, TaKaRa) according to the manufacturer’s instructions. Each tissue was biologically replicated three times using different samples. Complementary DNA (cDNA) synthesis was performed with a cDNA synthesis kit (HiScript® III 1st Strand cDNA Synthesis Kit, Vazyme) according to the manufacturer’s instructions, and qRT-PCR was performed using a kit (Taq Pro Universal SYBR qPCR Master Mix, Vazyme) according to the manufacturer’s instructions. The tomato gene *elongation factor 1 alpha* (*EF1α*) was selected as an internal reference gene, and all analyses included three technical replicates. Each sample was investigated in 3 biological replicates, and the 2^-ΔΔCt^ method was used to calculate the relative expression of genes in different samples [48]. The relevant primer sequences are listed in Table S1.

### Subcellular localization analysis

The full-length *SlSRM1-like* gene was cloned using tomato cDNA as a template and primers (forward: 5′-GAATTCATGACAGTAGATAAATCAAGA-3′; reverse: 5′-GTCGACAGGTTGGTCGTAGTAATATC-3′) with *Eco*RI and *Sal*I restriction sites. Both the PCR product and a pYBA1132 vector plasmid were digested with *Eco*RI and *Sal*I and then ligated via T4 DNA ligase after gel recovery and purification. The recombinant products were transformed into *Escherichia coli*, which was then cultured on Luria-Bertani (LB) solid media supplemented with kanamycin. After positive selection and sequencing, the correct plasmid was amplified and transformed into *Agrobacterium* GV3101, and *Agrobacterium* was subsequently collected by centrifugation and diluted with the infection solution (which consisted of 10 mmol/L MgCl_2_, 50 mmol/L 2-(N-morpholino)ethanesulfonic acid (MES) and 100 μmol/L acetosyringone to an optical density (OD) value equal to approximately 1.0 for injection into the underside of leaves of *Nicotiana benthamiana* plants that were approximately 28 d of age. After the tobacco plants were cultivated in the dark, the epidermis was removed, dyed with DAPI, and sectioned for observation under a fluorescence microscope.

### Obtaining and detecting mutants

The specific sgRNAs used for CRISPR/Cas9 vector construction were designed using the CRISPR-P 2.0 website (http://crispr.hzau.edu.cn/CRISPR2/) [49]. The corresponding primers were designed based on sgRNAs and synthesized for PCR. PCR was completed using the pCRM vector as the template, and the product was purified and digested with *Bsa*I and then purified again according to the instructions provided with the gel recovery kit. The plant expression vector pKTCR was digested with *Bsa*I, purified, and then ligated with the target fragment using T4 DNA ligase. The recombinant vector was transformed into *Escherichia coli* DH5α, and the *Escherichia coli* was cultured on LB solid medium containing kanamycin. The colonies were sequenced after the identification of positive clones by PCR. The correctly sequenced colony was amplified and then used to extract the plasmid using the kit.

Ailsa Craig tomato seeds were sterilized with 10% NaClO solution and sown on 1/2 Murashige and Skoog (MS) medium. When the cotyledons were fully expanded, they were cut into small pieces and precultured for 2 d. The recombinant vector was transformed into *Agrobacterium tumefaciens* GV3101 and then used to infect the precultured cotyledons. The cotyledons were cocultured in the dark for 2 d and then transferred to callus medium. Substitution was performed every 2 weeks until green shoots appeared. The buds were then transferred to the bud induction medium for continuous cultivation and then to the rooting medium to obtain transgenic plants [50]. All the tissue culture materials were cultured in an incubator at 25 °C with 1800 lx and a 16-h light/8-h dark cycle. All media formulations are shown in Table S2.

The leaves of the transgenic plants were used for the extraction of DNA by the hexadecyl trimethyl ammonium bromide (CTAB) method. PCR was accomplished with marker gene-specific primers. Transgenic plants with T-DNA successfully inserted into the genome were subjected to target sequence and off-target sequence mutation detection by PCR and sequencing. The successfully edited plants were propagated through self-pollination. The homozygous offspring that were successfully edited without T-DNA insertion were self-propagated for the next experiment.

All primer syntheses and sequencing were completed at Beijing Genomics Institution (BGI, Shenzhen, China), and all the primer sequences are shown in Table S3.

### Paraffin section experiment

The leaves of the plants grown for 45 d were cut and immersed in formalin-aceto-alcohol (FAA) solution. The processed samples were first immersed in different concentrations of alcohol (30, 50, and 70%, respectively, for 1 h; 85, 95, 100, and 100%, respectively, for 30 min) for dehydration. After the samples were soaked in a mixed solution (v:v = 1:1) of xylene and alcohol for 20 min, they were soaked in xylene for 1 h, which was then replaced with new xylene, and the samples were soaked for another 40 min. Then, the materials were immersed in 25, 50, and 75% paraffin-xylene solution and pure paraffin (twice) and incubated at 40 °C for 30 min each time. The fully soaked samples were then transferred to a paper boat filled with paraffin placed on cold water. After trimming, the paraffin blocks were sliced with a microtome, and successive slices were placed on glass slides. The dried slices were immersed in xylene, xylene and alcohol mixed solution (v: v = 1: 1), alcohol, 95% alcohol, 85% alcohol, safranine (4 h), 95% alcohol, solid green (30 s), 95% alcohol (30 s), alcohol, xylene and alcohol mixed solution (v: v = 1: 1), and xylene (twice) for 5 min. Gum was dripped onto the glass slides, the cover glass was applied, and then the slides were placed in a 42 °C incubator to dry. Successful sections were observed using a microscope.

### Measurement of leaf size

When the plants had grown for 35 days and 45 days, the leaves on the bottom branches were selected for leaf size measurement. Ten different plants were randomly selected for statistical analysis.

### Phylogenetic analysis

Genes homologous to the *SlSRM1-like* (LOC101245350) gene were queried in the Kyoto Encyclopedia of Genes and Genomes (KEGG) database (https://www.kegg.jp/kegg/genes.html). The sequences of the top 100 proteins with highly similar sequences were downloaded and used to construct a phylogenetic tree. The phylogenetic tree was completed using the neighbor-joining (NJ) method in MEGA X software, and 1000 bootstrap replications, the Poisson model and pairwise deletions were used. The iTOL website (https://itol.embl.de/) was used for postmodification and hygienics of the phylogenetic tree.

### Statistical analysis

Significance analysis of the data was performed using SPSS software. Student’s *t* test was used to analyze the significance of the leaf size and the qRT-PCR results.

## Supplementary Information

**Additional file 1: Figure S1.** Off-target prediction analysis of sgRNA target sites. **Figure S2.** Identification of T-DNA insertions in mutant tomato plants. **Figure S3.** PCR identification after CRISPR vector construction. **Figure S4.** Vectors and basic procedures for CRISPR experiments. **Figure S5.** Photos of paraffin slices of tomato leaves.

**Additional file 2: Table S1.** Sequences of primers used for qRT-PCR. **Table S2.** Formula of the medium used in the tissue culture process. **Table S3.** Primer sequence used to obtain transgenic plants.

## Data Availability

Not applicable.

## Abbreviations

SlSRM1-like: *Solanum lycopersicum* salt-related MYB1-like
CRISPR/Cas9: Clustered, regularly interspaced, short palindromic repeat/CRISPR-associated 9
*SlSRM1-like*-M: *SlSRM1-like* mutant
*PIN1*: *PIN-formed 1*
IAA: Indoleacetic acid
*LA*: *Lanceolate*
*LYR*: *Lyrate*
*PTS*: *Petroselinum*
*RAX1-like*: *Regulator of axillary meristems1-like*
*Tf*: *Trifoliate*
BLAST: Basic Local Alignment Search Tool
GFP: Green fluorescent protein
sgRNAs: Small guide ribonucleic acids
Kan: Kanamycin
PCR: Polymerase chain reaction
T-DNA: Transfer DNA
*AS1*: *Asymmetric leaves 1*
*AS2*: *Asymmetric leaves 2*
NCBI: National Center for Biotechnology Information
LB: Luria-Bertani
OD: Optical density
MS: Murashige and Skoog
CTAB: Hexadecyl trimethyl ammonium bromide
FAA: Formalin-aceto-alcohol
KEGG: Kyoto Encyclopedia of Genes and Genomes
NJ: Neighbor-joining
