# Supplementary material for: CRISPR-Cas9-mediated mutagenesis of the SlSRM1-like gene leads to abnormal leaf development in tomatoes
Source: BMC Plant Biol. 2022 Jan 3;22:13. doi: 10.1186/s12870-021-03397-5 (PMC8722279; doi:10.1186/s12870-021-03397-5)
Supplement: Supplementary file 1 — Additional file 1: Figure S1. Off-target prediction analysis of sgRNA target sites. Figure S2. Identification of T-DNA insertions in mutant tomato plants. Figure S3. PCR identification after CRISPR vector construction. Figure S4. Vectors and basic procedures for CRISPR experiments. Figure S5. Photos of paraffin slices of tomato leaves. [file 12870_2021_3397_MOESM1_ESM.docx]

Yao Tang, Figure S1

A
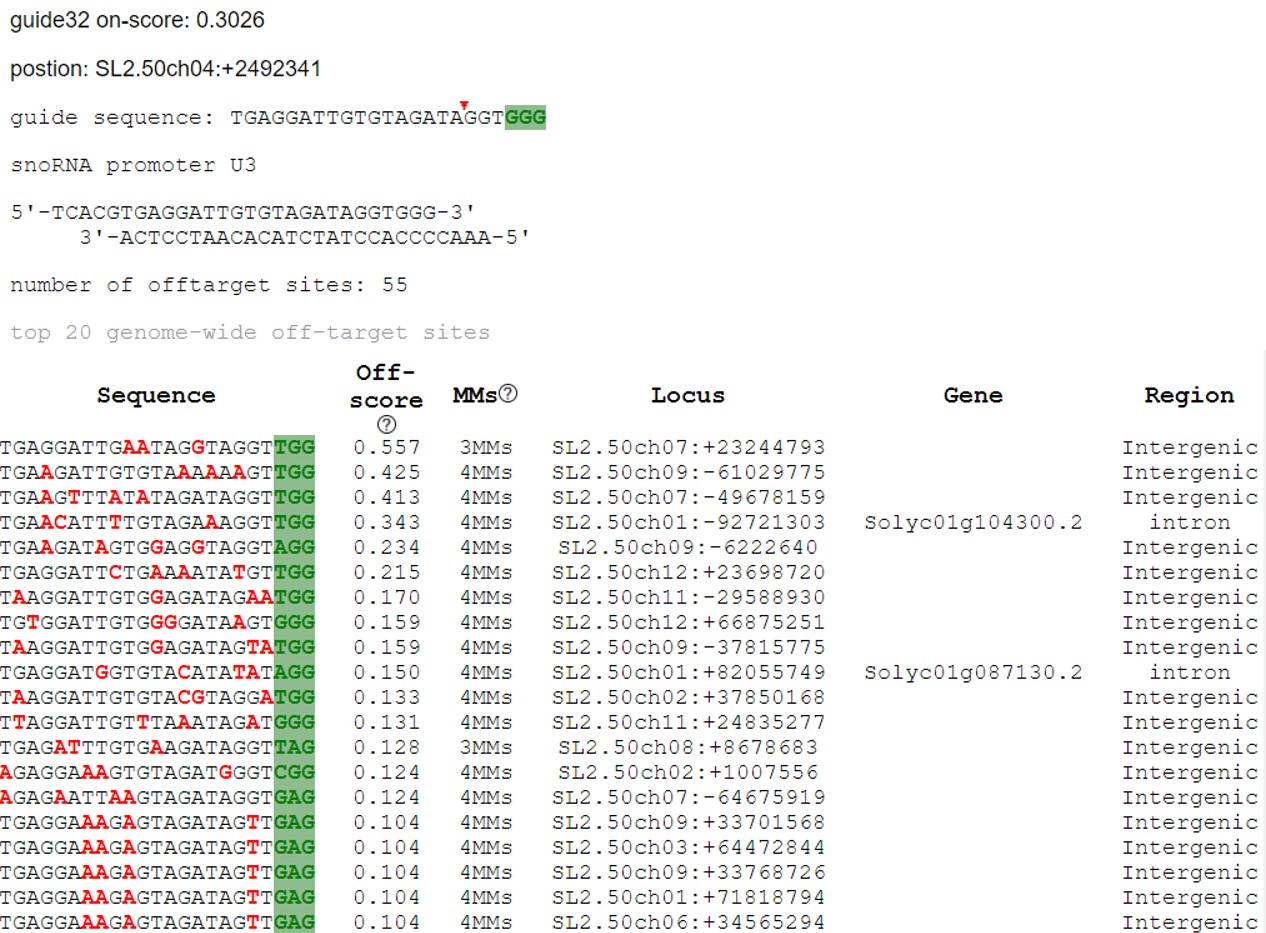


B
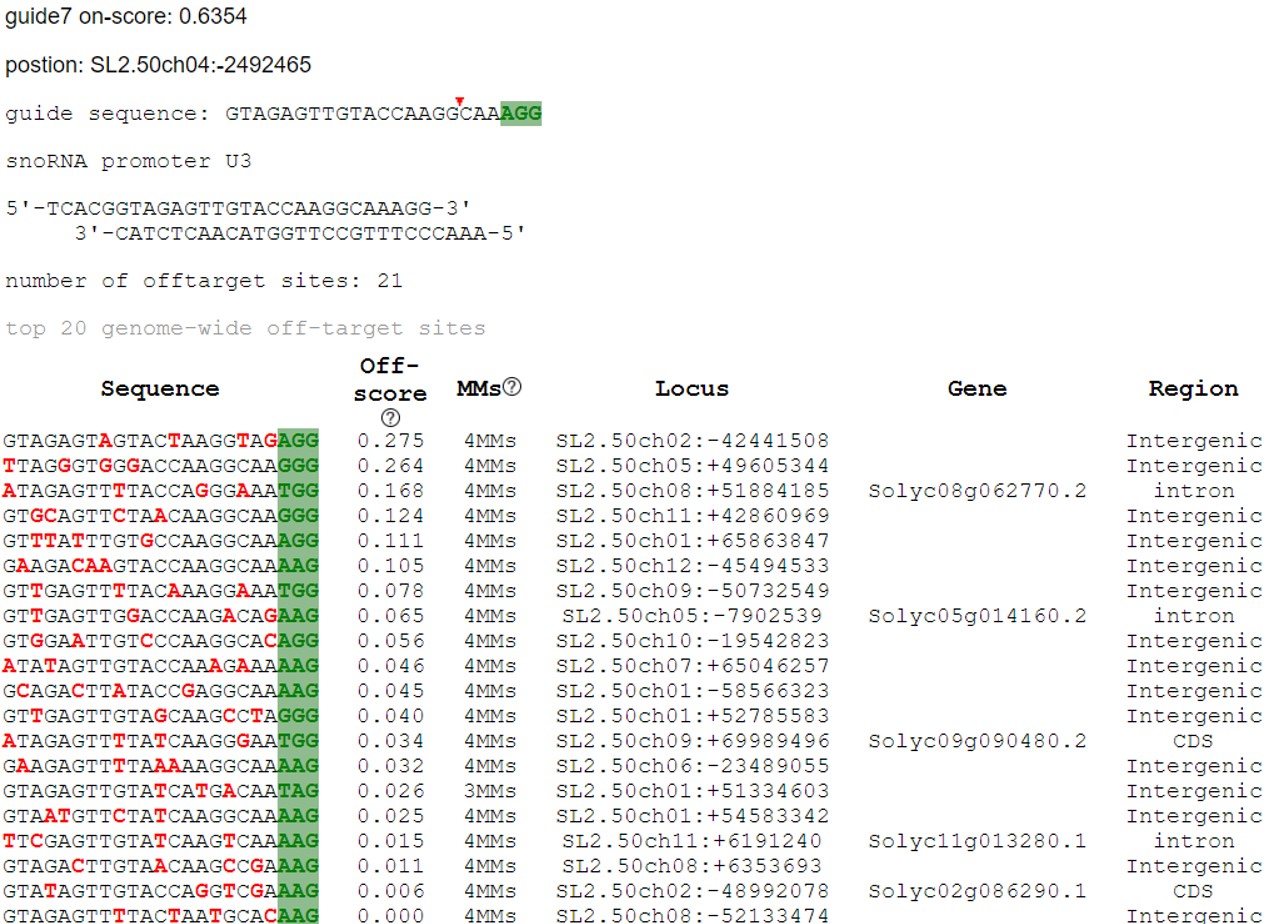


Off-target prediction analysis of sgRNA target sites. The results were predicted by the CRISPR-P 2.0 website. The figure shows only the top 20 most likely off-target sites.

Yao Tang, Figure S2


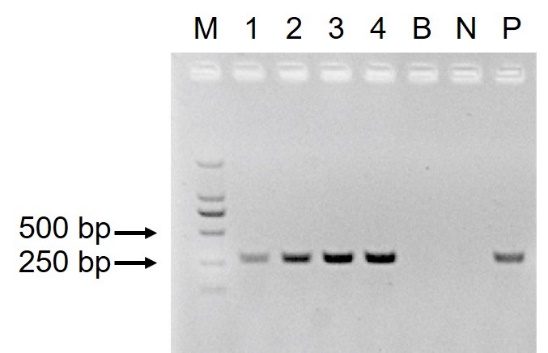


Identification of T-DNA insertions in mutant tomato plants. Primers for the *NPTII* gene were used for detection, and the PCR product was 289 bp. Nos. 1-4 are the results of tomato plants edited by CRISPR/Cas9. M, marker (2000 bp). B, blank control. N, negative control. P, positive control.

Yao Tang, Figure S3


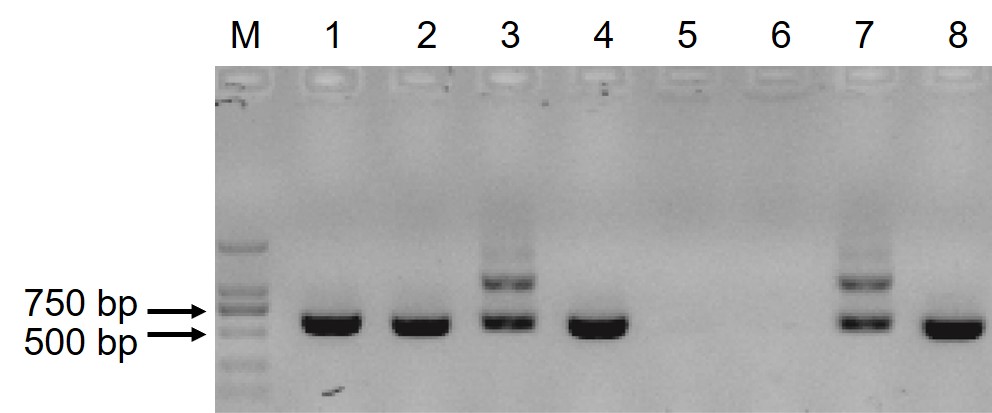


PCR identification after CRISPR vector construction. Nos. 1-8 are the PCR detection results using different templates. M, marker (2000 bp).

Yao Tang, Figure S4


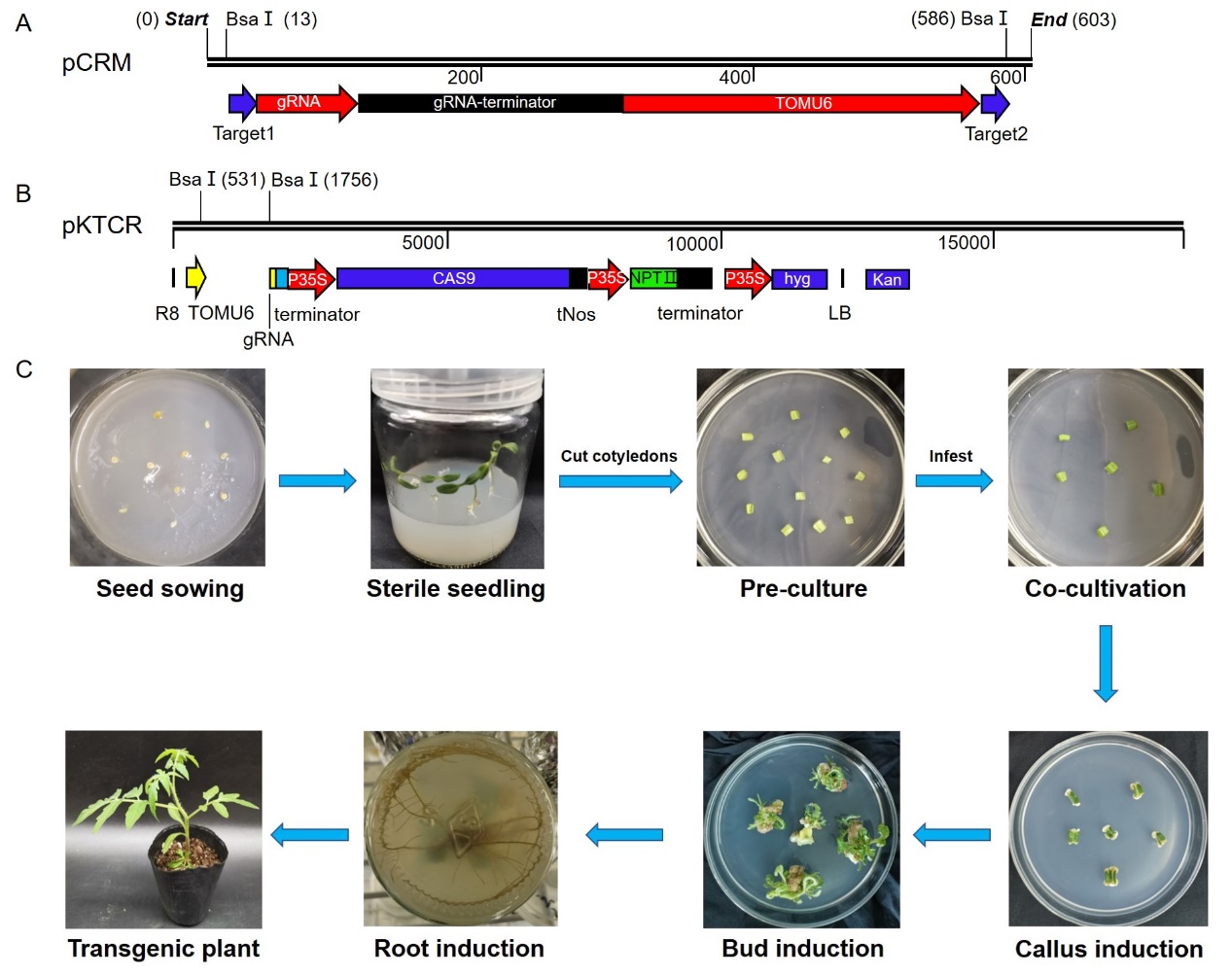


Vectors and basic procedures for CRISPR experiments. (A) Basic diagram of the pCRM vector. (B) Basic diagram of the pKTCR vector. (C) The basic process of the CRISPR experiment.

Yao Tang, Figure S5


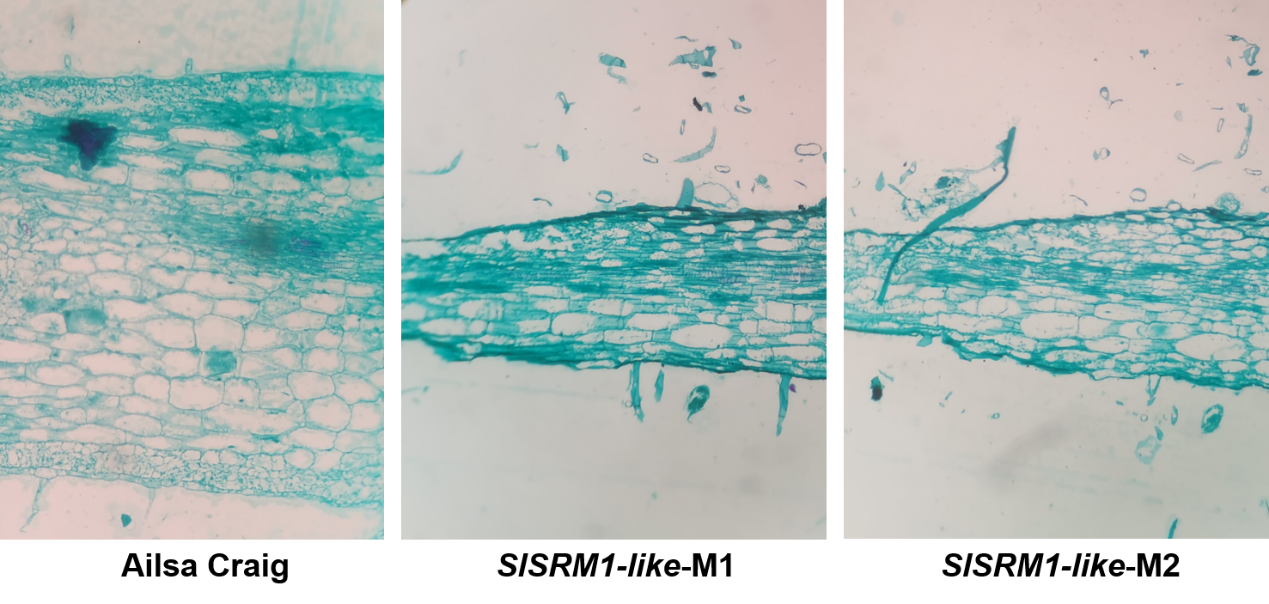


Photos of paraffin slices of tomato leaves. Tomato plant leaves grown for 45 d were used as material for paraffin sections. The photos were taken at a magnification of 400.
