# Supplementary material for: CRISPR-Cas9-mediated mutagenesis of the SlSRM1-like gene leads to abnormal leaf development in tomatoes
Source: BMC Plant Biol. 2022 Jan 3;22:13. doi: 10.1186/s12870-021-03397-5 (PMC8722279; doi:10.1186/s12870-021-03397-5)
Supplement: Supplementary file 2 — Additional file 2: Table S1. Sequences of primers used for qRT-PCR. Table S2. Formula of the medium used in the tissue culture process. Table S3. Primer sequence used to obtain transgenic plants. [file 12870_2021_3397_MOESM2_ESM.docx]

Yao Tang, Table S1 Sequences of primers used for qRT-PCR

| Gene names | Forward primers (5’-3’) | Reverse primers (5’-3’) |
| --- | --- | --- |
| *LA* | TCAAAAAGGCAAAACCCGCC | ACTTGAAGGGCCAGCAACAT |
| *LYR* | ACTTGGGGGACACATGAACC | AAACCATACGCGGTGGAGAG |
| *PTS* | AAGATAGGAGCTCCGCCAGA | TTCTATCCATTGCCTCGCCC |
| *Tf* | ATGGACCAAATGGCTCACCC | TGCCTATCCCAAGATCTGCC |
| *RAX1-like* | TACCAGGGAGGACCGACAAT | TGGACTAGTGGAATGGGCTC |
| *AS1-like* | CGAGCTTGGGCAGACCATAA | GCGGTCTAATCTGCAACCCA |
| *AS2-like* | AAATGAGTTGCAGCCACACC | GAGATGACACCAACGCAACC |
| *URL1-like* | ACATAGCGATGAGCGGTGAG | GGTGAACAGTGCTGCCAATG |
| *ROC5-like* | GAAGGCTTCCTTCTGGCTGT | AGCTTCAACATGCTACGCCT |
| *PIN1* | CCAGCCTGCAGCACCTAATA | TGAGGCCAAACAAGCTCGAA |
| *PIN3* | CCCACGAGGGTCGAATTTCA | GGCCTCCGATACTGGTGAAG |
| *IAA3* | GCTAAGATTAGGTTTGCCTGGG | TTTTGGTGCAGGAGCTGAGT |
| *IAA9* | ATTCCGGTGTGAAAGCAGGT | ACCTGTGCCTTTGTAGCAGG |
| *ARF3* | TGTTCCTGTGACGCTGATGT | TCCTGAGACGAGAGCTCCTAC |
| *ARF4* | TGATAAACCAGTCCGCCCAC | AGGTTTTCCCCAAGCACCAA |
| *EF1α* | CCACCAATCTTGTACACATCC | AGACCACCAAGTACTACTGCAC |

Yao Tang, Table S2 Formula of the medium used in the tissue culture process

| Media | Composition |
| --- | --- |
| 1/2 MS medium | 1/2 MS + 15 g/L agar + 15 g/L sucrose |
| Pre-cultured medium | MS + 15 g/L agar + 30 g/L sucrose + 0.2 mg/L IAA |
| Bacteria suspension medium | MS + 20 g/L sucrose + 100 mg/L Myo-inositol + 100 mg/L Thiamine HCl |
| Co-cultured medium | MS + 15 g/L agar + 30 g/L sucrose + 2.0 mg/L 6-BA + 0.2 mg/L IAA |
| Callus medium | MS + 15 g/L agar + 30 g/L sucrose + 0.2 mg/L IAA + 2.0 mg/L 6-BA + 50 mg/L Kan + 50 mg/L Carb |
| Bud induction and elongation medium | MS + 15 g/L agar + 30 g/L sucrose + 0.2 mg/L IAA + 2.0 mg/L 6-BA + 2.0 mg/L ZT + 50 mg/L Kan + 50 mg/L Carb |
| Rooting medium | MS + 15 g/L agar + 30 g/L sucrose + 0.1 mg/L NAA + 50 mg/L Kan + 50 mg/L Carb |

Yao Tang, Table S3 Primer sequence used to obtain transgenic plants

| Primer name | Primer sequence (5’-3’) | Function description |
| --- | --- | --- |
| Target 1 | TGAGGATTGTGTAGATAGGT | Target site sequences |
| Target 2 | GTAGAGTTGTACCAAGGCAA |  |
| pCRM-T1-F | ATATATGGTCTCGTTTGGAGGATTGTGTAGATAGGTGTTTTAGAGCTAGAAATAGC | Cloning target site sequence and positive identification after constructing the vector |
| pCRM-T2-R | ATTATTGGTCTCGAAACTTGCCTTGGTACAACTCTACCAAACTACACTGTTAGATTC |  |
| pKTCR-F | CGACGGCCAGTGCCAAGCTTA | Sequencing after completing the vector construction |
| pKTCR-R | TGCAGGCATGCAAGCTTATTG |  |
| *NPTⅡ*-F | ACTGGGCACAACAGACAATCG | Detecting the presence of T-DNA |
| *NPTⅡ*-R | GCATCAGCCATGATGGATACTTT |  |
| *SRM1-like*-F | ATGACAGTAGATAAATCAAGAAGCTC | Sequencing at the target sites |
| *SRM1-like*-R | GAGGTGCTCGACCACTTGA |  |
| Off-target 1-F | GAAGGCAATCATCCCCGACT | Sequencing at the off-target site 1 |
| Off-target 1-R | ATGCGGGTCGTATACGTGTG |  |
| Off-target 2-F | CACGTTCAATTACGCGTGCTC | Sequencing at the off-target site 2 |
| Off-target 2-R | AAATCCACAACGTCCCCCAA |  |
